# Experimental Models of In Vitro Blood–Brain Barrier for CNS Drug Delivery: An Evolutionary Perspective

**DOI:** 10.3390/ijms24032710

**Published:** 2023-01-31

**Authors:** Bivek Chaulagain, Avinash Gothwal, Richard Nii Lante Lamptey, Riddhi Trivedi, Arun Kumar Mahanta, Buddhadev Layek, Jagdish Singh

**Affiliations:** Department of Pharmaceutical Sciences, School of Pharmacy, College of Health Professions, North Dakota State University, Fargo, ND 58105, USA

**Keywords:** blood–brain barrier (BBB), endothelial cells (ECs), in vitro models, microfluidics, neurotherapeutics, transwell model

## Abstract

Central nervous system (CNS) disorders represent one of the leading causes of global health burden. Nonetheless, new therapies approved against these disorders are among the lowest compared to their counterparts. The absence of reliable and efficient in vitro blood–brain barrier (BBB) models resembling in vivo barrier properties stands out as a significant roadblock in developing successful therapy for CNS disorders. Therefore, advancement in the creation of robust and sensitive in vitro BBB models for drug screening might allow us to expedite neurological drug development. This review discusses the major in vitro BBB models developed as of now for exploring the barrier properties of the cerebral vasculature. Our main focus is describing existing in vitro models, including the 2D transwell models covering both single-layer and co-culture models, 3D organoid models, and microfluidic models with their construction, permeability measurement, applications, and limitations. Although microfluidic models are better at recapitulating the in vivo properties of BBB than other models, significant gaps still exist for their use in predicting the performance of neurotherapeutics. However, this comprehensive account of in vitro BBB models can be useful for researchers to create improved models in the future.

## 1. Introduction

Neurological disorders are responsible for the most disability cases globally and are the second leading cause of death worldwide. CNS disorders account for 16.5% of global deaths as of 2016 [1]. Therefore, the need to develop therapies against central nervous system disorders is imminent from a clinical, social, and economic standpoint. However, on analysis of the trend, it is observed that therapies targeted against CNS disorders have among the lowest approval rates with prolonged approval periods [2]. Since the 1980s, only 10–12% of drugs have been approved for CNS disorders per decade globally, and require, on average, 20% more time than non-CNS drugs. This scant approval of drugs can account for multiple factors ranging from inadequate understanding of the disease pathology, lack of proper animal models, and ambiguous clinical trial endpoints [3]. One of the significant causes contributing to this shortage of CNS drug pool is the absence of in vitro blood–brain barrier (BBB) models mimicking the endogenous barrier properties. The potential therapeutic candidates directed towards CNS disorders are prescreened for their ability to cross the BBB prior to animal testing or clinical trials. Therefore, the neuropharmaceutical industry has been searching for ideal barrier models to rapidly and efficiently screen drug candidates for clinical trials [4]. 

Given the underlying challenges in solving this paradox of BBB for therapeutic targeting, historically, the complete understanding of this barrier and its location posed a formidable challenge to experts. In 1885, for the first time, Paul Ehrlich performed a classic experiment to comprehend the nature of BBB. He injected acidic vital dye intraperitoneally into animals and found that almost all organs in the body were stained except the brain and spinal cord. Ehrlich attributed this phenomenon to the differential binding affinity of dyes to different organs. One of Ehrlich’s students, Paul Goldman, later injected the dye trypan blue (MW 960 Da) into the CSF and surprisingly found staining in the brain, contrary to the tissue binding hypothesis Ehrlich proposed. Goldman also injected the dye intravenously and reported a lack of staining in the brain. These observations led to the understanding that the brain is compartmentalized from other organs [5]. After studying the penetration of numerous molecules into the brain and CSF, Lina Stern and Raymond Galtier in 1921 coined the term “barrière hémato–encéphalique” (i.e., blood–brain barrier) [6]. Later, Brightman and Reese, through their seminal paper published in 1969, unambiguously proved the anatomical location of BBB in cerebral capillary endothelium through the permeability of horseradish peroxidase (HRP), 40 KDa protein [7]. 

In vivo techniques are the gold standard for screening drug candidates. They represent the complexity of the BBB and even provide a platform to study the effect of treatments on cellular, tissue, and organ levels. Additionally, animal models allow the exploration of various pharmacodynamic, pharmacokinetic, and immunological parameters. Despite all these benefits, the in vivo BBB models are expensive and time-consuming to operate and involve rigorous ethical norms. Reports show that more than 80% of drugs shown to be effective in animal models eventually fail at clinical trials. This failure can be subjected to the poor correlation of genetically modified animal physiology to humans and species variation in expression profiles of some key proteins, and substandard methodology and regulation of animal experiments [8]. At the same time, the role of other factors such as inappropriately designed preclinical trials, ambiguous end point targets, and irrational selection of patient population in the failure of clinical trials cannot be overlooked [9,10]. Additionally, real-time measurement of analytes for mechanistic studies using animals is limited by its complex physiology and difficulties in the experimental procedure [11]. As a result, there arises a need for in vitro BBB models that are robust, easy to fabricate and analyze, offer better in vitro/in vivo correlation, and can handle a large number of samples for high-throughput screening. Therefore, in this review, we detail the trajectory of the development of different in vitro BBB models and discuss each model extensively. 

## 2. Blood–Brain Barrier (BBB): A Detailed Account

BBB is a diffusional interface between the brain tissue and the blood vasculature essential for the homeostasis and functioning of the brain [12]. The lack of an energy storage mechanism in the brain necessitates the uninterrupted supply of nutrients through the vasculature around the clock. On top of that, being a highly energy demanding organ, the brain consumes 15–20% of total oxygen from the cardiac output as well as 15–20% of total glucose. As a result, to compensate for this high energy demand in a continuous fashion, the brain has ample distribution of blood capillaries with a total surface area of 15–25 m^2^. These blood capillaries in the brain are essentially different from the rest of the capillaries in two ways. First, they form a tight barrier regulating the transport across the brain, and second, they exhibit plastic properties, i.e., constriction or dilation of capillary diameter in response to abnormal physiological conditions [13]. In the following section, we dive into the nature, properties, and function of the barrier existing in the brain capillaries. 

### 2.1. Components of BBB

**Figure 1:**
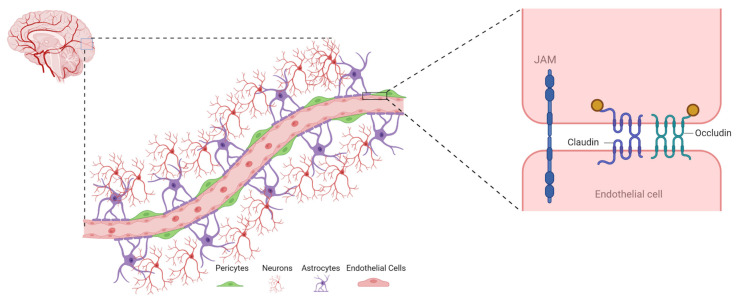
Schematic representation of structural components of the blood–brain barrier (BBB) and junction protein in endothelial cells. The endothelial cells on brain capillaries are ensheathed by foot process of astrocytes and closely associated with pericytes, which together form a neurovascular unit. Structural components in endothelial cells responsible for barrier integrity are shown on the right, i.e., claudin, occludin, and JAM (Created with BioRender.com).

The structural unit of BBB comprises microvascular endothelial cells (ECs), astrocytes, pericytes, and the basement membrane (Figure 1). Together these units form a close-knit assembly referred to as a neurovascular unit [14]. Here, we discuss the different cellular and acellular components of BBB.

#### 2.1.1. Endothelial Cells (ECs)

The brain microvascular ECs are the building blocks of BBB, which are situated at the blood and the brain interface. Endothelial cells in the mature mammalian brain have different characteristics compared to the other ECs in the body. The brain ECs have a flattened appearance, consist of several caveolae at the luminal surface, have a higher mitochondria population, and include inter-endothelial tight junction proteins [15]. Moreover, pore size in the peripheral capillaries is generally 6–7 nm, while brain capillaries are tighter (50–100 times), restricting the permeability of hydrophilic solutes [16]. The greater number and volume of mitochondria enhance the energy potential and are believed to be important criteria for the active transport of nutrients to the brain [17]. 

The development of BBB from the endothelium starts during embryonic angiogenesis and continues up to adulthood through intimate association with the other components of the neurovascular unit. The ECs are involved in different kinds of biological activities of the brain, such as transportation of macro- and micronutrients, osmoregulation, leukocyte trafficking, and receptor-mediated signaling. 

The structural components of the ECs, which are mainly responsible for the above properties, are as follows: (1) tight junction proteins (e.g., occludin, claudin), (2) adherent junction proteins (VE-cadherin and E-cadherin), and (3) junctional adhesion molecules (JAM) [17]. The first two of these proteins form the backbone of tight junction strands, and the third one helps in immune surveillance and inflammatory action by trafficking neutrophils, dendritic cells, and T-lymphocytes from vascular compartments and lymphoid.

Occludin is known as the first discovered membrane protein within the tight junction proteins. The molecular weight of the occludin is around 60~65 KDa and has three cytoplasmic domains, four transmembrane domains, and two extracellular domains. N-terminus and C-terminus are related to the intracellular part [18]. Occludin has the potential to bind with the C-terminus to F-actin, which makes it unique compared to the other membrane proteins. C-terminus also has the ability to bind with the GK (guanylate kinase) domain of zona occludens (ZO), as a result of which the GK domain becomes localized into the cell membrane [19]. The truncation of the C-terminus and N-terminus of occludin leads to a decline in TEER, confirming occludin’s role in the tight junction barrier function [18]. 

Claudins belong to the family of 24 proteins having a molecular weight of 20–40 kDa. Claudins also contain four transmembrane domains, a short carboxyl intracellular tail, and two extracellular loops [19]. Claudins participate in forming the backbone of the tight junction with the help of extracellular loops by forming dimers and binding homotypcially to other claudin molecules present in the ECs [20]. Claudins bind to the PDZ (PSD-95/discs large/zonula occludens-1) domain of zona occludens through the C-terminus of intracellular loops. Claudins in different forms are found in brain ECs such as claudin-1, -2, -3, -5, -11, and -12 [20]. Among different kinds of claudins, claudin-5 is a hallmark of BBB and helps in the early-stage angiogenesis of the central nervous system [21]. Claudin-5 is found to express exclusively in the brain ECs as well as in the blood vessels of the lungs and kidneys [22].

Junctional adhesion molecules (JAMs), members of the immunoglobulin superfamily, have a molecular weight of 40 kDa and contain three structural domains, a short intracellular tail, a single transmembrane domain, and two immunoglobulin-like loops [12]. JAMs are categorized into JAM-A, JAM-B, JAM-C, JAM-4, and JAML [23]. Among all JAMs, JAM-B and JAM-C are found in ECs, whereas JAM-A is found in the cerebrovessels [24]. The stability of the tight junction is maintained by JAM-A, JAM-C, and JAM-B in combination [25].

#### 2.1.2. Astrocytes

Astrocytes are a type of glial cells that cover >99% of the endothelium of BBB [26]. The interaction between astrocytes and ECs plays an important role in the development of BBB. This interaction also enhances the EC tight junctions, thereby reducing the gap between the junctional area [27]. Astrocytes participate in various biological functions of the brain other than BBB regulation, such as neurotransmitter and ion uptake/recycling, synapse formation, maintaining potassium levels in the extracellular matrix, and controlling the inflammatory responses in CNS [28]. They ensheath neuronal processes and blood vessels, ensuring neuronal metabolism, neurotransmitter uptake, and regulation of ion concentration within the extracellular space. In addition to these functions, astrocytes repair the CNS following an injury, regulate vascular tone in response to neuronal activity, and maintain the BBB phenotype [29].

#### 2.1.3. Pericytes

Pericytes are also highly abundant in CNS. Brain capillaries are made of pericytes having different frequencies in different vascular beds. Connexins and N-cadherin in pericytes form a close association with endothelium by forming peg–socket focal contact points [15]. Pericytes exhibit various biological functions in association with the ECs, such as the exchange of metabolites, ions, ribonucleic acid, and secondary messengers. They also play an important role in maintaining BBB integrity, microvascular stability, and angiogenesis. Contractile characteristics of pericytes, such as smooth muscle cells, help to regulate the capillary diameter and cerebral blood flow [30]. Numerous reports suggest the presence of receptors for vascular mediators such as endothelin-1, angiotensin-1, vasopressin, catecholamines, and vasoactive intestinal peptides in pericytes [15]. 

#### 2.1.4. Basement Membrane

The basement membrane, which is mostly underestimated, is an acellular member of the neurovascular unit and an active component of the BBB. It comprises complex extracellular matrix molecules such as perlecan, osteonectin, fibronectin, agrin, and glycosaminoglycans generated by Ecs and astrocytes [31]. The basement membrane anchors cells and supports BBB integrity, helps in intercellular communication, and maintains the polarized features of the components of the neurovascular unit.

### 2.2. Functions of BBB

#### 2.2.1. Protection against Neurotoxins

BBB acts as a natural shield and protects the delicate neuronal environment from circulating toxins due to its physiological barrier function. These toxic substances could either be endogenous metabolites or proteins, externally administered xenobiotics, or environmentally acquired agents [15]. Additionally, various ATP binding cassettes, i.e., ABC transporters on Ecs, continuously efflux these toxic agents to maintain a toxin-free environment in the brain [32].

#### 2.2.2. Prevention of Macromolecules Influx into the Brain

The protein content of cerebrospinal fluid (CSF) is lower compared to plasma due to the filtration of protein by the choroid plexus [15]. Plasma proteins such as albumin, prothrombin, or plasminogen are harmful to neural tissues as they lead to cellular activation and, eventually, apoptosis. Infiltration of these proteins into the brain leads to the conversion of prothrombin to thrombin by factor Xa present in the brain. Similarly, the tissue plasminogen activator in the brain activates the plasminogen to plasmin. The presence of plasmin or thrombin initiates a series of events leading to seizures, glial cell activation, and cell death [32]. Thus, BBB serves to prevent an influx of these macromolecular proteins from plasma to avoid any undue damage to the brain. 

#### 2.2.3. Regulation of Neurotransmitters

The central and peripheral nervous systems share some identical neurotransmitters. Thus, BBB acts as a barrier between these two separate neurotransmitter pools to avoid any crosstalk that could originate unintended neuronal responses [32]. One such example is that of the neuroexcitatory molecule glutamate. Glutamate level increases sharply in blood after the meal, and if the entry of this compound is unrestricted through BBB, it might lead to permanent neuroexcitatory damage to the brain [33]. BBB thus plays an important role in compartmentalizing the body’s neurotransmitter pool.

#### 2.2.4. Maintenance of Ionic Homeostasis

Ions and their homeostasis are vital contributors to the generation and propagation of nerve signals. BBB acts as a key regulator of ionic composition in CSF. Through ion pumps such as Na^+^ K^+^ ATPase, BBB maintains the homeostatic balance of ions such as Na^+^, K^+^, Ca^++^, or pH levels in the brain [32]. There is a variation in these ionic levels in plasma following exercise or a meal, but BBB ensures the ionic balance remains unperturbed in the brain even after these activities [34]. 

#### 2.2.5. Supplementation of Brain Nutrition 

BBB, by its tight endothelial junctions, limits the paracellular transport of essential nutrients such as glucose, vitamins, and amino acids into the brain. However, Ecs in BBB house transmembrane-specific receptors such as GLUT (glucose transporter) and LAT (L-type amino acid transporter) that help in the transport of these water-soluble nutrients and ensure the brain is supplied with the essential nutrients [35].

## 3. Transport across BBB

**Figure 2:**
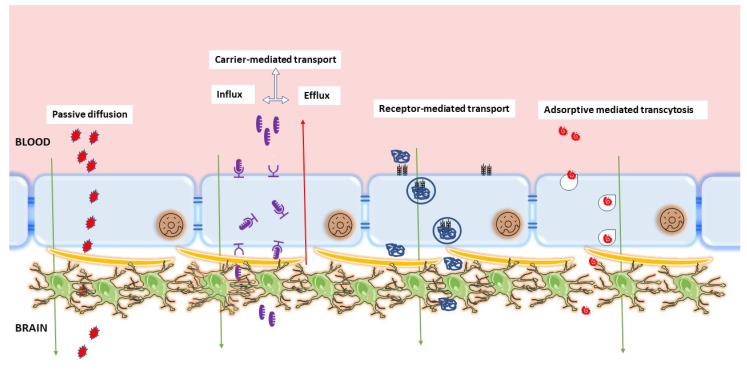
Major routes of transport across the BBB. The figure describes passive diffusion, carrier-mediated transport, receptor-mediated transport, and adsorptive-mediated transcytosis as major routes of transport across the endothelial layer of brain. The figure was inspired by Dwivedi, Divya, Kanu Megha, Ritwick Mishra, and Pravat K. Mandal. “Glutathione in brain: overview of its conformations, functions, biochemical characteristics, quantitation and potential therapeutic role in brain disorders”. *Neurochem. Res.* **2020**, *45*, 1461–1480 [37].

The BBB has unique structural, transport, metabolic, and adhesion properties that differentiate brain Ecs from non-neuronal Ecs. These characteristic properties promote and strengthen the formation of AJ, TJ, and TEER barriers, which severely impedes paracellular diffusion across the BBB. Some molecules can pass through the BBB despite these active inhibitory properties. Several studies have explored the true nature and complexity of the BBB in an attempt to elucidate the mechanisms behind its protective effects and passage of selective materials across the BBB [36]. Here, we discuss some of the few proposed ways of material transport across the BBB (Figure 2). 

### 3.1. Passive Diffusion

Although the BBB is stringent in reducing influx into the brain, certain substances readily make it across the BBB. However, only molecules fitting specific profiles can cross the BBB. A key determinant of candidates for passive diffusion is lipophilicity. Generally, highly lipophilic molecules readily diffuse across the BBB passively [38]. A strong correlation exists between the rate at which a substance enters the brain and its lipid solubility and can be determined from the substrate’s logP (i.e., octanol/water partition coefficient at pH 7.4) [15]. In addition to lipid solubility, substances that diffuse across the BBB usually have poor hydrogen bonding capacity; in other words, highly polar substances do not permeate the BBB easily [39]. Generally, molecules with more than eight hydrogen bonds rarely cross the BBB in therapeutically relevant amounts. Another proposed property of molecules that cross the BBB is their charge at a certain pH. In general, possessing a positive charge at pH 7–8 favors brain permeation, while having a negative charge at pH ≤ 5 disfavors brain permeation [40,41]. Finally, the ability of a molecule to diffuse across the BBB is determined by its size, shape, and flexibility. While it is generally accepted that flexible molecules rapidly diffuse across the BBB, the relationship between shape and BBB diffusion is inconclusive. While some studies concluded that spherical-shaped molecules more easily diffuse the BBB, another study concluded that increasing molecular branching decreases BBB permeation [42,43]. However, once the molecular weight exceeds 400 Da, the permeability of the molecule is no longer determined by its solubility in lipids. The passage of small hydrophobic molecular compounds is projected to be through transitory pores in the phospholipid bilayer. The pore is believed to be created by a normal molecular motion as the free fatty acyl side chains kink within the phospholipid bilayer [15,44,45]. 

### 3.2. Active Efflux

The BBB plays a significant role in maintaining brain homeostasis by preventing harmful endogenous and exogenous substances from entering the brain. The abundance of ATP-binding cassette efflux on the luminal, blood-facing endothelial plasma membrane of the BBB mainly ensures these pharmacoresistant characteristics of the BBB. Efflux pumps in the BBB serve as gatekeeper detoxification systems within the brain [46,47]. Certain lipophilic molecules that are suitable substrates of various efflux transporters can be easily transported out of the brain by the ATP-driven efflux pumps. Active efflux pumps prevent substances from entering the brain in an energy-dependent, unidirectional, outwardly directed transport against the substrate concentration gradient [47,48]. The ABC efflux transporter P-glycoprotein, multidrug resistance protein, and breast cancer resistance protein (BCRP) significantly contribute to BBB efflux activity. These efflux activities ensure no or minimal neurotoxic effects of drugs that can otherwise easily penetrate the BBB. Efflux activity is substrate-specific, and although the molecule entering the brain is a suitable candidate for the efflux pumps, it will not be transported out of the brain. In other words, weak substrates of BBB efflux transporters can evade these transporters and retain within the brain. 

Albeit helpful in protecting the brain, efflux transporters have become a barrier to brain delivery of therapeutic molecules. Efflux pumps are known to be overexpressed in some neurological disorders, such as epilepsy, where they limit management success through resistance to medications [49]. More recently, ABC transporters have become maneuver targets to increase the distribution of drugs into the brain [49].

### 3.3. Carrier-Mediated Transport (CMT)/Influx by the Major Facilitator Superfamily

The brain requires a constant supply of energetic substrates not synthesized within itself and a means to eliminate their metabolites [50,51]. While it is relatively easy for lipophilic substrates to diffuse into the brain passively, polar or hydrophilic compounds require some help to enter the brain [52,53]. In humans, specific proteins, which are products of gene superfamilies, mediate the transport of specific nutrients through biological membranes, including the BBB [54,55]. Within the brain, major facilitator superfamily transporters have been identified, including Mfsd2a, and solute carriers (SLC1), among others.

Solute carriers are expressed in different regions of the BBB where they serve specific functions. The BBB ECs’ solute carriers ensure the absorption of essential substrates from the blood [56]. In neurons and glial cells, solute carriers maintain brain homeostasis and drug response regulation [54,57], while solute carriers expressed in the choroid plexus of the blood–cerebrospinal fluid barrier regulate secretion and re-absorption of the cerebrospinal fluid [58]. Examples of substrates that enter/exit the brain through the solute carrier-mediated transport include sugars (such as glucose, galactose, fructose, and mannose), amino acids (such as glutamine, alanine, serine, and threonine), folates, urea, and citrates [54].

### 3.4. Receptor-Mediated Transport (RMT)

Due to their physicochemical properties, some macromolecules required by the brain (such as transferrin and insulin) cannot directly cross the BBB. The transfer of these molecules across the brain is facilitated by receptor-mediated transcytosis (RMT) [59]. The receptors for these ligands are situated on the luminal surface of the BBB, where they shuttle substances in and out of the BBB. RMT occurs when the ligand binds to the receptor and triggers ligand–receptor complex endocytosis. The complex is routed through various compartments in the late endosome where the cargo is detached, the macromolecule is liberated on the abluminal side of the cell, and the receptor is either recycled back to repeat the cycle or delivered to late endosomes and targeted for lysosomal degradation [58]. Three types of endocytic vesicles are known to be involved in macromolecule trafficking. These vesicles are (1) clathrin-coated pits, which are involved in most RMT processes at the BBB; (2) caveolae, which participate in adsorptive-mediated endocytosis of extracellular molecules and receptor trafficking; and (3) macropinocytotic vesicles [60,61]. RMT has been widely exploited for brain-targeted delivery of both drugs and genes [62,63]. The most widely studied targets for RMT are transferrin [62,64,65], insulin receptor, and low-density lipoprotein receptor [60].

## 4. Embryonic Development of BBB

BBB formation is mediated by cellular and noncellular component interaction with ECs, which can be grouped into three overlapping stages: angiogenesis, differentiation, and maturation [66]. Despite the general understanding of several pathways involved in BBB formation, some details still elude scientific knowledge. Fortunately, since BBB development is evolutionarily preserved, it has paved a path to understanding human BBB development through animal models over the past decade [67]. Here, we discuss the formation of the BBB as far as recent knowledge permits. 

BBB formation begins during early embryogenesis, before astrocyte generation, and in the perineural vascular plexus surrounding the neural tube. The initial stages of BBB formation are characterized by sprouting angiogenesis-driven neuroepithelial vascularization. These early sprouts possess BBB properties. Sprouting angiogenesis is often directed by molecular growth signals, including bone morphogenetic proteins (BMP), vascular endothelial growth factor (VEGF), fibroblast growth factor (FGF), and Wnt secreted by neural progenitor cells in the embryonic brain. These factors are often present in a concentration gradient (increasing towards the hindbrain) which drives the process of angiogenesis. During angiogenesis, endothelial progenitor cells from the perineural vascular plexus invade the neural tissue and enter the neuroepithelium in a pattern similar to angionenesis. 

In addition to directing sprouting angiogenesis, Wnt plays a crucial role in BBB formation, chiefly inhibiting the degradation of β-catenin in the proteasome, resulting in the accumulation of β-catenin. Accumulation of β-catenin promotes its translocation into the nucleus, where it induces the transcription of target genes, without which vessel formation in the central nervous system remains incomplete. Wnt also directly induces the expression of genes, including those encoding for tight junction formation and nutrient transporters such as Glut-1 (encoded by Slc2a1). Recently, an orphan member of the G protein-coupled receptor family, GPR124, has also been identified to play a critical role in brain-specific angiogenesis. GPR124 promotes ligand-specific canonical Wnt signaling ensuring proper survival, growth, and migration of brain ECs [66].

Brain EC migration in sprouting angiogenesis is coupled with the recruitment of pericytes by secretory cues arriving from the ECs. These cells secrete platelet-derived growth factor B (PDGF-BB), which recruits pericytes and ensures their proliferation. PDGF-BB further promotes migration and attachment of pericytes to the cerebral endothelium within a day of the angiogenic invasion. Recruited pericytes embed in a typical basement membrane within the BBB ECs, exhibiting long cytoplasmic processes that cover the endothelium [68]. The pericytes seal the embryonic BBB in a leak-preventing fashion and continue to do so even in maturation. Despite not directly impacting the expression of tight junction proteins, pericytes are vital for suppressing transcytosis and leukocyte adhesion molecule expression [69,70]. These actions equally ensure the restriction of substance trafficking into the brain. Pericytes also participate in vessel formation, remodeling, and stabilization during angiogenesis and continue to regulate neurovascular function into adulthood. Pericyte interaction with brain ECs results in the expression of the transforming growth factor-β (TGF-β) receptor and its ligand on both cells. Secretion of TGF-β results in the production of cadherin-2 and the subsequent secretion of different extracellular matrix components, which contribute to basement membrane formation. Another factor that promotes the proliferation and migration of ECs is the VEGF-A. The neural tube expresses VEGF-A like all other factors in a gradient that induces capillary growth towards the higher concentration (neuroectoderm). The expression of VGF-A is driven by a hypoxic microenvironment within the developing embryo [71]. The formation of this vasculature is key to the establishment of the BBB.

Till this point, the premature BBB layer is weak in restricting the passage of substances across itself. Brain ECs express tight junction proteins (occludin and claudin-5) to strengthen the barrier further by sealing the gaps inhibiting selective movement of solutes across the epithelium. Rudimentary strands of tight junction proteins have been observed on the evading ECs [71], suggesting that this expression occurs at the initiation of angiogenesis as the ECs invade the CNS [72,73]. In addition to the tight junction proteins, high levels of leukocyte adhesion molecules are expressed on invading endothelial cells. Leukocyte adhesion molecules promote the transfer of inflammatory molecules by encouraging large amounts of transcytosis, making the BBB seemingly leaky.

Astrocytes hold the pericytes onto the ECs and form the astroglial feet. During this period, the tight junction proteins become more complex and decrease vesicle trafficking across the barrier. In addition to the sonic hedgehog, astrocytes secrete apolipoprotein E (ApoE), angiopoietin (Ang-1), and angiotensinogen (Agt), which also enhance the expression of BBB phenotype such as overexpression of efflux pump proteins.

The evolution of the BBB phenotype characterizes BBB differentiation. The establishment of complex tight junctions and the evolution of transport systems for hydrophobic compounds alter the premature layer of ECs, pericytes, and astrocytes into a multifaceted BBB. At this point, pericytes surrounding the brain ECs secrete different factors, including TGF-B, which repress the leaky properties of the brain ECs. The factors also lower the expression of leukocyte adhesion molecules and plasma lemma vesicle-associated protein, effectively lowering the trafficking of leukocytes and different plasma molecules into the brain.

BBB maturation is achieved through the expression of tight junction proteins and their subsequent redistribution throughout the entire BBB. Wnt signaling between brain ECs and astrocytes regulates tight junction protein production. The combination of the tight junction sealing and inhibition of transcytosis by the pericytes are the greater contributors to the role of the BBB. Further, the inhibitory effect of tight junctions is complemented by the overexpression of P-glycoprotein in the BBB. P-glycoproteins function as efflux pumps that effectively return substances that make it across the barrier into the bloodstream. 

The complete BBB is defined by unique structural, transport, metabolic, and adhesion properties that differentiate ECs and their organization in the brain from the non-neural tissues. In summary, a fully mature BBB will have ECs surrounded by pericytes, a basement membrane that plays a protective role and regulates the flow of charged macromolecules and astroglial feet, which maintain the BBB properties. Tight junction proteins (claudins, occludin, and zona occludens) between brain ECs create a physical barrier, sealing the paracellular route and creating a high transendothelial cell electrical resistance (TEER), which further impedes ions and small charged molecules from crossing the BBB. Adherens junctions also exist at the site of endothelial cell-to-cell contacts. VE-cadherin dimers in adherens junctions help in intercellular adhesion in the cell membrane and attach to the actin cytoskeleton by catenins. 

## 5. In Vitro Models of BBB

Over the years, various in vitro models have been developed incorporating diverse design strategies to replicate the brain’s barrier properties. BBB studies started in 1953 with monolayer cell culture on transwell systems. As the isolation of primary cerebral microvessels was pioneered in the 1970s, the in vitro BBB incorporated these cells to study brain permeation. The knowledge of the formation of tight junction proteins using ECs and astrocytes surfaced in the 1980s, giving rise to co-culture transwell models. In the 1990s and 2000s, transwell co-culture using three different cell types was also used with greater emphasis on transendothelial electrical resistance (TEER) value as a measure of barrier integrity. The late 2000s and early 2010s saw the emergence of dynamic and 3D models such as organoids or barriers on chips using microfluidics. Modern-day models have improved the microfluidic design and focused on the vasculogenesis approach to mimic the BBB as closely as possible [74].

### 5.1. 2D Models of BBB

Conventional in vitro models are composed of isolated primary brain ECs or immortalized cells, which are used as a monolayer in the 2D BBB models [75]. These models are generally used to evaluate transportation/permeability across the BBB. In these models, cells are cultured on inserts that facilitate TEER measurement and permeability assessment. Selection of the cells for luminal and abluminal surfaces (primary vs. immortalized, source species) is critical because it determines overall effectiveness and usability. In general, 2D models offer advantages such as being cost-effective, highly convenient for high-throughput screening, and enhanced barrier tightness [76].

#### 5.1.1. Cells Used in 2D Models

The reliability of an in vitro model depends on how closely it mimics the physiological features of the BBB in situ, including tight junction sealing, and the expression of various efflux proteins and receptor molecules [77]. There are different types of cells used for BBB formation, i.e., primary, immortalized, and stem cells. Generally, primary cells are preferred because they resemble their in situ counterparts [78]. However, the major limitations of using primary cells are their limited availability and high cost [79]. Particularly for human cells, the availability of brain tissue is limited and solely depends on the clinical collaborator. Additionally, specific skill sets are required to isolate the cells from the human brain tissues. Poor cell viability and differentiation of primary cells are other issues in maintaining these cells. Herein, we describe two types of cells frequently used for in vitro BBB development. 

##### Endothelial Cells

The ECs isolated from the brain capillaries are commonly used to develop in vitro BBB models. However, a co-culture of ECs with astrocytes, pericytes, or neurons better mimics an in vivo BBB environment [80]. Primary ECs also have some favorable properties for constructing the BBB models, including closely resembling in vivo BBB phenotype [81,82]. Brain ECs constructed in vitro BBB models are among the most successful due to higher TEER and low permeability [83]. Still, there are some limitations associated with primary brain ECs, including difficulty in maintenance, high cost, the requirement of particular skill sets, limited availability, time-consuming procedure, and high risk of contamination [82,84,85]. 

Additionally, immortalized ECs have been used in in vitro BBB model preparation as an alternative to primary ECs. Available literature confirms the notion of increased numbers of studies using immortalized cells based in vitro BBB model. More than 35 types of immortalized ECs were used in different documented studies [86]. The most commonly used immortalized ECs are the human microvascular EC line hCMEC/D3, rat EC line RBE4, and mouse brain microvascular EC line bEnd.3 [86]. However, there is a lack of comparative studies using various cell types. Immortalized ECs are also associated with some drawbacks, including low baseline TEER [87,88,89] and poor tight junction protein expression [87]. Thus, stem cells could be a better cell source for constructing scalable and reliable models that closely resemble in vivo BBB systems. Table 1 lists different types of ECs used in the in vitro BBB model development. 

##### Stem Cells

Initially, primary and immortalized brain ECs were used to prepare in vitro BBB models [90]. Stem cell-based in vitro BBB models were introduced to overcome the inherent limitations in developing in vitro BBB models using primary and immortalized cells. Stem cells can differentiate into brain ECs and be a reliable cell source for the in vitro human BBB models [16]. Different types of stem cells have been used in BBB modeling, i.e., embryonic stem cells, induced pluripotent stem cells, and mesenchymal stem cells. 

Stem cells are derived from fetal brains (human or mice) and used in stem cell therapy because these cells can differentiate into all types of brain cells and self-renew [91]. Neural stem cells can differentiate into neuronal cells, and mesenchymal stem cells have similarities with pericytes so that they can be used in in vitro BBB models [92]. However, some complex ethical limitations are associated with embryonic stem cells due to adverse impacts on the human embryo [93]. Additionally, stem cells differentiation involves inherent issues such as reproducibility in differentiation protocol, separation of endothelial cells from heterogeneous cell mixture, and achieving the target phenotype representative of endothelium [94].

#### 5.1.2. Monoculture BBB Model 

Monoculture BBB models are simple in vitro models with a single layer of brain ECs in the transwell insert. In these models, the well and insert mimic blood and the endothelial wall, respectively. Lower passage cells are generally used to mimic the unique properties of brain ECs. Some limitations are associated with monoculture models, including the use of only one major type of cell and the absence of other cell types. Consequently, monoculture models lack cross-talk of ECs with astrocytes or pericytes, which plays a crucial role in maintaining the BBB properties [95,96,97].

**Table 1:** Endothelial cells used in in vitro BBB models and their source.

Cell Types	Origin	References
Immortalized Cells		
bEnd.3 cells	Mouse	[98,99,100]
cEND	Mouse	[101]
BB19	Human	[102]
hBMEC	Human	[102]
bEnd.5	Mouse	[103,104]
cerebEND	Mouse	[101]
ECV304	Human	[105]
CRL-2583	Mouse	[106]
EaHy929	Human	[107]
HCEC	Human	[108]
hCMEC/D3	Human	[109,110,111,112,113]
TY10	Human	[102]
TY08	Human	[114]
TR-BBB	Rat	[114,115]
TM-BBB	Mouse	[116]
THBMEC	Human	[117]
HUVEC-304	Human	[118]
Primary Cells		
BMEC	Bovine	[119]
BCEC	Bovine	[120,121]
PBEC	Porcine	[122]
Brain capillary endothelial cells	Monkey	[84]
Stem Cells		
Induced pluripotent cells	Human	[94,123,124,125]
Induced multipotent cells	Human	[125]

#### 5.1.3. Co-Culture BBB Models

The co-culture in vitro BBB models are considered one of the simplest and most feasible models, composed of a brain EC monolayer under a static culture environment with astrocytes or pericytes cultured additionally. In the past decade, multicellular BBB models have been introduced using brain ECs and other brain cells, including pericytes and/or astrocytes [69,126,127]. Multicellular BBB models with the addition of neurovascular cells have proven their applicability in drug screening and beyond [128]. However, brain ECs with astrocytes and pericytes are extensively used as they offer paracellular tightness in the BBB and implicate EC’s functions [83,126].

In vitro BBB models prepared with primary brain ECs offer a better understanding of BBB’s cellular and molecular mechanisms. Co-culture of BBB-associated cells, such as astrocytes, that mimics in vivo conditions of the BBB is more relevant [129]. Astrocytes contribute tight junction characteristics to BBB [130]. Available literature suggests that using astrocytes in BBB preparation leads to a robust and stable model with high TEER. Abbott et al., 2012, prepared a BBB model using rat brain ECs and neonatal astrocytes [129]. Similarly, the incorporation of glial cells ensures superior integrity and recapitulates in vivo BBB environment [131]. Glial cells play a crucial role in nutrient uptake, establishing the first diffusion barrier. These co-culture models also form septate junctions to restrict paracellular diffusion [132]. A recent study prepared an in vitro BBB model by co-culturing bEnd.3 cells and glial cells [133]. The developed BBB with high TEER (178.4 ± 10 Ωcm^2^) was intact and restricted the paracellular transport. The researcher used the developed BBB to investigate the targeting efficiency of the transferrin and cell-penetrating peptide-anchored liposomes across it [133]. Even higher TEER (>300 Ωcm^2^) has been claimed with a similar co-cultured BBB model [134]. Table 2 lists different cells used to prepare co-culture BBB models.

Pericytes and ECs are the closest neighbors in vivo but are less characterized than the astrocytes. Pericytes are responsible for the production of laminin, collagen (type IV), and glycosaminoglycans. Unlike astrocytes, pericytes have angiogenic, immune, phagocytic, and contractile functions [135]. A line of reference is available to acclaim the permeability regulation ability of the pericyte. Apart from the permeability, gene expression also has been reported in endothelial/pericyte cell co-culture, i.e., angiopoietin-1-induced Tie-2-mediated occludin gene and ABC transporter expression. Pericytes offer reduced permeability of the BBB over astrocytes [95]. However, several studies have also shown BBB-deteriorating effects of pericytes [136] and increased in vitro BBB permeability [137]. These reports explain higher heterogenicity in pericytes’ multiple functional states.

**Table 2:** Commonly used cells in in vitro co-culture BBB models.

Cells	Inference	Reference
Human induced pluripotent (hiPSC) and multipotent cells (neural stem cells)	High TEER (2500 Ωcm^2^); complex in vivo-like tight junction network	[125]
iPSC and GM25256 cells	High TEER (1560 Ωcm^2^ ± 230 Ωcm^2^); TJ protein and endothelial marker expression	[138]
Human hCMEC/D3	Feasible alternative to primary ECs in in vitro BBB models	[139]
bEnd.3 and glial cells	Intact BBB model with 178.4 ± 10 Ωcm^2^ TEER	[133]
bEnd.3 and glial cells	TEER ~400 Ωcm^2^	[134]
bEnd.3 and glial cells	TEER ~600 Ωcm^2^	[63]
ECs and pericytes	Occludin gene expression	[95]
Human ECs and pericytes	TEER ~3500 Ωcm^2^	[140]
Bovine ECs and rat astrocytes	TEER ~850 Ωcm^2^	[119]
Porcine ECs and rat astrocytes	TEER ~1693 Ωcm^2^	[122]
Monkey ECs and rat astrocytes, pericytes	TEER~150 Ωcm^2^	[84]

**Figure 3:**
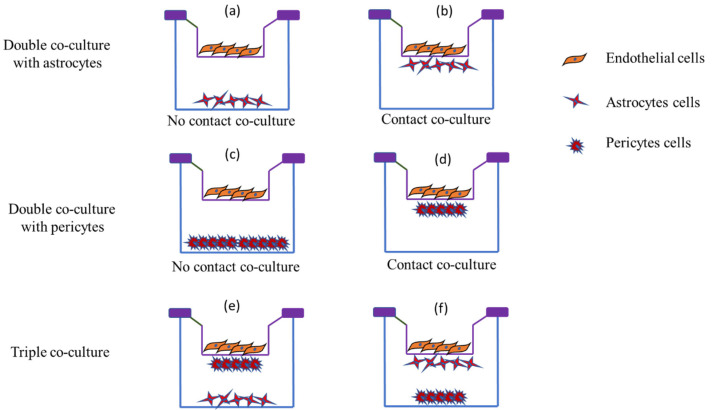
Schematic representation of different in vitro co-culture models in BBB demonstrating double co-culture and triple co-culture. Endothelial cells, astrocytes, and pericytes are the cells utilized for barrier formation. (**a**,**c**) represent no contact co-culture of endothelial cells with astrocytes and pericytes, respectively, while (**b**,**d**) represent endothelial cell contact co-culture with astrocytes and pericytes, respectively, and (**e**,**f**) represent triple co-culture where abluminal cells in contact with endothelial cells differ. (**e**) represents pericytes in contact with endothelial cells with astrocytes at the bottom of the cell, whereas (**f**) represents astrocytes in contact with endothelial cells and pericytes at the bottom of the well.

Crucial roles of pericytes and astrocytes in the endothelial modulations triggered triple co-culture BBB models. Figure 3 shows that there can be multiple possibilities for localizing three different cell types. In one of the initially developed BBB models, pericytes and astrocytes were cultured in the bottom of the inserts and wells, respectively (Figure 3e), which offers the best barrier properties [141]. In a different setup, the ECs were cultured on the bottom while mixed pericytes and astrocytes were on the top, offering higher TEER (300 Ωcm^2^). This in vitro BBB model is commercially available with monkey brain ECs [142]. The presence of primate ECs in the barrier makes it closer to the human in vivo system. Further, neural precursor cells were introduced with rat ECs and astrocytes, and the developed in vitro barrier showed higher TEER and increased P-gp protein levels [143].

#### 5.1.4. In Vitro Characterization of 2D BBB Models

##### In Vitro Permeability Measurement

The development and assessment of the integrity of the BBB during maturation and experimentation are critical. One of the most frequently used methods of permeability assessment is based on the transport of the tracer molecules across the BBB (Table 3). Numerous dyes have been used to investigate the permeability of in vitro BBB systems, such as sodium fluorescein (NaF), Evan’s blue, and horseradish peroxidase. Sodium fluorescein is a low-molecular-weight, non-toxic marker widely used in BBB permeability investigations [144]. NaF is detectable at very low concentrations, and due to its low molecular weight, it can easily extravasate the BBB. In addition, it does not bind to protein, meaning that the presence of NaF in the brain tissues depends on BBB permeability exclusively. Therefore, NaF could be a better low-molecular-weight marker than the protein-binding dyes [145]. High-molecular-weight markers are also available to determine BBB permeability, such as horseradish peroxidase (40 kDa) [146], dextran (286 Da to 2000kDa) [147], and Evan’s blue–albumin (69 kDa) [148]. 

The permeability of 2D models is determined using the equation below [80].

P_app_ = dQ/dt. 1/A. C_0_. 60 (cm/s),

where

P_app_ = apparent permeability;
dQ/dt = amount of permeability marker transported per min (μg/min);
A = surface area of the transwell membrane (cm^2^);
C_0_ = initial concentration of the permeability marker;
60 is the conversion factor.

The apparent permeability calculated of the permeation molecule using the above equation is referred to as P_t_. Similarly, the permeability of cell free systems is calculated and referred to as P_f_. The permeability of the system is then calculated using the following equation.

1/P_e_ = 1/P_t_ − 1/P_f_ (cm/s)

##### TEER Measurement

TEER measurement has been frequently used as one of the standard techniques to investigate the integrity of the BBB since its first introduction [152]. It measures electrical resistance across the barrier and reflects ionic transport. In contrast, the permeability coefficient (flux of non-electrolyte tracers) reflects the paracellular water flow and pore size [153]. Different methods are used for the TEER measurement: (1) electric impedance spectroscopy and (2) direct current or quasi-direct current methods. 

Electric impedance spectroscopy is mainly helpful for leaky or low-resistance in vitro barrier models. It provides detailed information regarding cells’ morphological changes and the tissue’s cellular construction [154]. Different devices are available to measure the impedance, including ECIS (electric cell substrate impedance sensing), xCELLigence, the hollow-fiber model, and cellZscope. These devices automatically measure the impedance spectra. On the other hand, quasi-direct current methods are used to measure micro-vessels in live tissues and brain endothelial monolayer cultures. The most used electrode types in the TEER measurements are chopstick electrodes, which are easy to handle. In this assembly, current electrodes are made of silver, and voltage-measuring electrodes are made of silver/silver chloride [155]. A hollow-fiber model is also available, with a bundle of porous membrane capillaries mimicking micro-vessels’ geometry [156].

### 5.2. Organoid Models

**Figure 4:**
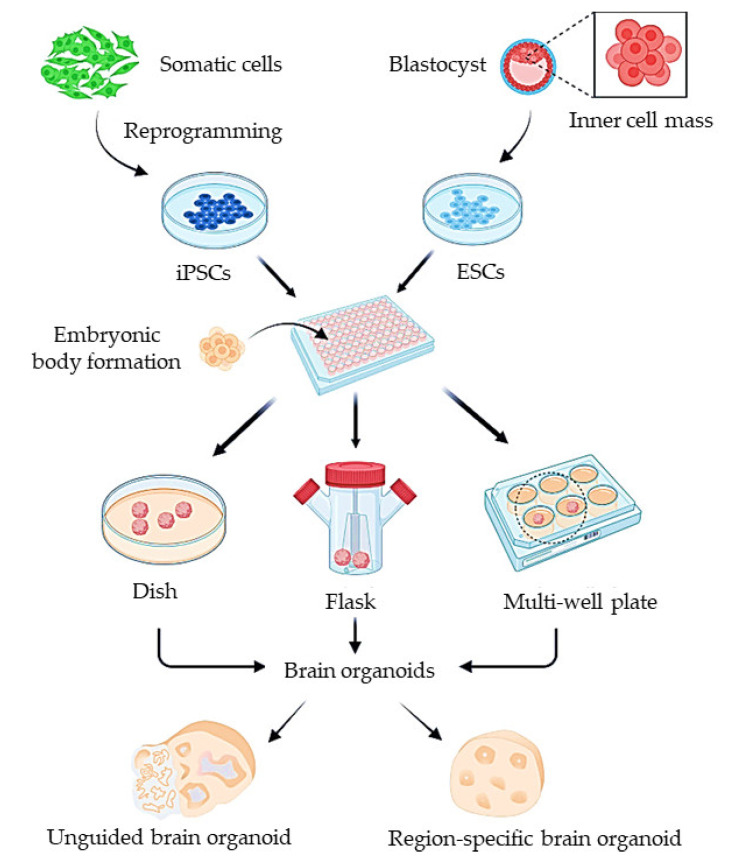
The generation of human organoids using induced pluripotent stem cells (iPSCs). Either iPSCs derived from somatic cells or embryonic stem cells (ESCs) are dissociated into single cells for generation of embryonic bodies (EBs). EBs are further cultured on dish, rotating flask, or multiple wells to form whole-brain organoids or region-specific organoids through guided differentiation. (Source: Reprinted from Lu, X.; Yang, J.; Xiang, Y. Modeling human neurodevelopmental diseases with brain organoids. *Cell Regen.* **2022**, *11*, 1–13, [165], under an open access Creative Commons CC BY 4.0 license).

The CNS has been difficult to model owing to the physiological barrier surrounding it. A proper understanding of the BBB transport mechanism and developing an effective BBB model would lead to better identification of CNS-targeting therapeutics or approaches to improve brain delivery. Consequently, this would significantly improve the development of therapies for neurodevelopmental and neurodegenerative disorders [157]. There are broad discrepancies in results obtained from 2D cell cultures and in vivo experiments. Animal models are heavily relied on for studies in neuroscience due to the complexity of neurological disorders. However, in the past few decades, 3D cell culture techniques have tremendously improved and can help bridge this gap and provide more realistic information compared to 2D models [158]. Organ spheroids or organoids are an invaluable tool for mimicking crucial features of the in vivo environment. The technique involving organoids has evolved from embryoid cultures where stem cells clustered in 3D and self-organize to form disparate tissues in vitro, analogous to teratoma formation in vivo [159]. The use of organoids as a BBB model can help us construct the proper structural architecture of the barrier similar to that observed in vivo. Organoids can help realistically model in vivo conditions and processes, which can provide in vivo-like responses [160]. The development of such models would facilitate researchers to identify better approaches for transporting CNS therapeutics across the BBB and predict whether new therapeutics can cross the BBB [157]. Organoids can be constructed with primary cells or from cells differentiated from embryonic or induced human pluripotent stem cells (Figure 4) [158]. 

BBB models constructed using primary cells are widely used owing to their accessibility and being well documented in the literature [161]. Primary cells collected from animals have drawbacks, such as not having the typical composition and function of human cells. For instance, they lack tight junction (TJ) protein expressions [162]. Though human primary cells express these characteristic TJ proteins, surprisingly present BBB models using human primary cells still have low TEER values (under 100 Ω/cm^2^) [163]. A 3D multicellular BBB organoid model has been developed using six different types of brain cells (astrocytes, ECs, pericytes, oligodendrocytes, neurons, and microglia cells) to determine the transport of ultrasmall gold nanoparticles [164]. The organoids had a diameter of around 500 μM and were impermeable to dextran FITC until the stimulation with mannitol was performed. The penetration of the fluorescent-labeled nanoparticles was studied using confocal microscopy [164]. 

Even though models constructed with primary cells are considered standard, these cells tend to lose their properties over time, which could lead to the model having weak barrier properties [166]. To surmount these shortcomings, induced pluripotent stem cells (iPSCs) have been employed to develop BBB models.

Human iPSCs have been employed in several different in vitro models since their development [167]. Even though numerous protocols describe the steps to produce brain microvascular endothelial cells (BMECs) from iPSCs, there are discrepancies in the protocol. However, the following steps were common in the protocol: iPSCs culture, endothelial induction, BMEC specification, and purification [168]. A major advantage of utilizing iPSCs for the organoid model is the ability of these cells to differentiate into different cells while maintaining genetic consistency, supporting the production of a patient-specific in vitro BBB organoid model [163]. These cells can be differentiated spontaneously to form undirected organoids or brain region-specific organoids by introducing signaling molecules to direct them to resemble particular brain regions [158]. Figure 4 represents the generation of brain organoids, both unguided and region-specific, to mimic the functional unit of brain that houses brain capillaries which can be employed for BBB studies. iPSCs make excellent barrier models due to their ability to express BBB markers (occludin, claudin-5, and ZO-1) and transporters such as GLUT-1 and P-glycoprotein. The permeability and TEER values of these organoid models (~351 Ωcm^2^) are equivalent to the values obtained from in vivo measurements [163,169]. These cells have a limited in vitro life span, involving complex and time-consuming processes of dedifferentiation and redifferentiation. In addition, iPSCs have tumorigenic and teratogenic potential; thus, the removal of differentiated potentially tumorigenic cells must be realized [163]. Patient-derived iPSCs can also be used to create neurological disease organoid models. Self-organizing 3D organoids were constructed from human neural tissues derived from iPSCs, and these organoids could mimic the Alzheimer’s disease phenotype. It was found that the treatment of these patient-derived organoids with β- and γ-secretase inhibitors considerably decreases amyloid and tau pathology [170]. A recent adoption in the cerebral organoid development technique is the utilization of a spinning bioreactor, which enables better oxygen and nutrient absorption. This supports the formation of large and complex organoids due to the formation of longer neuroepithelium zones [171]. A miniaturized spinning bioreactor has been developed to generate forebrain, midbrain, and hypothalamus-specific organoids from human iPSCs. The organoids were modeled for zika virus exposure, and antiviral drug penetration was tested [159].

### 5.3. Microfluidic Models for BBB 

The application of microfluidic devices in BBB design started in the early 2010s [172]. Before the advent of microfluidic technology, dynamic in vitro models were developed to improve the static models that were extensively utilized at the time [173]. Although dynamic in vitro models introduced fluid flow into the system, their application was limited shortly after due to thicker walls and minimal interaction with astrocytes or pericytes. 

Microfluidics essentially deals with the behavior and manipulation of fluids at micron-sized channels. Structures ranging from channels, valves, or chambers with dimensions comparable to those seen in tissues or organs are constructed utilizing advanced engineering and materials such as silica, glass, quartz, or macromolecular polymers [174]. When cells are cultured in these channels with continuous perfusion with the appropriate media, they give rise to the functional unit of tissues or organs that replicate their functions. The microfluidic devices can be customized to the required use, from a single cell type to multiple cells in complex designs. Additionally, fluid flow, shear stress, or mechanical strain can be modified to study tissue or organ-specific functions [175]. The biological functions derived from these devices are more accurate than computer-based models or extrapolated data from animal testing. These devices use minimal materials to fabricate, are portable, and are relatively cheaper options for studying organ functions [176].

#### 5.3.1. Designing of Microfluidic Devices

The design of microfluidic devices for BBB should be performed with four considerations: (1) ECs lining the lumen-like structure; (2) cellular interactions between ECs, astrocytes, and pericytes; (3) dynamic fluid flow with shear stress; and (4) thin basement membrane of around 100 nm in thickness. Photolithography using polydimethylsiloxane (PDMS) or 3D printing techniques is commonly used to fabricate microfluidic devices. The integrity of microfluidic BBB is ensured either by determining the level of tight junction markers such as occludin or claudin in the assembly or by measuring the permeability using sodium fluorescein or dextran. TEER measurement is not always feasible to evaluate the barrier’s integrity, as placing electrodes is cumbersome in this assembly. 

##### Sandwich Design

**Figure 5:**
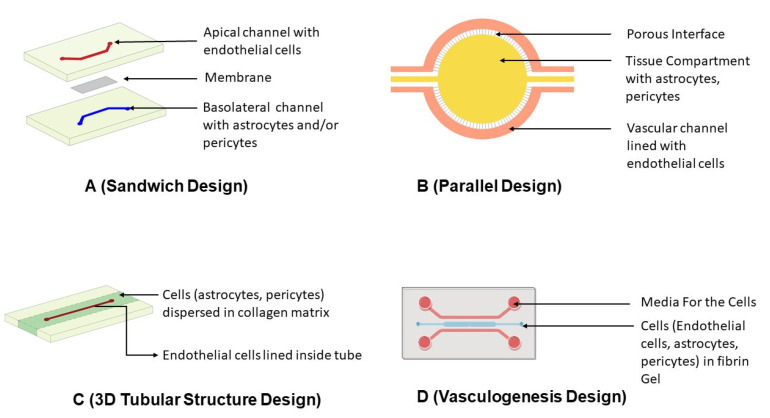
Schematic diagram of the design strategies adopted in the fabrication of microfluidic chip-derived in vitro BBB models (**A**) Sandwich Design: Upper and lower PDMS channel separated by a membrane. The apical channel is plated with endothelial cells, whereas the basolateral channel consists of astrocytes and/or pericytes. (**B**) Parallel Design: This consists of two parallel horizontal channels with perforations in the inner channel that provide contact between them. The outer channel has endothelial cells, whereas inner channel consists of astrocytes or pericytes dispersed in matrix. (**C**) 3D Tubular Structure Design: This consists of central cylindrical channel in collagen matrix. The central cylindrical channel has endothelial cells, whereas the matrix has astrocytes or pericytes dispersed in matrix. (**D**) Vasculogenesis Design: This consists of the central channel having endothelial cells, astrocytes, or pericytes dispersed in matrix flanked by two channels nourishing the media.

For designing the first-generation microfluidic BBB models, the primary focus was to include the added functionality of fluid flow lacking in previous designs. As a result, the first kind of microfluidic device was fabricated by adopting the concept from a 2D transwell design called a sandwich model (Figure 5). It consisted of upper and lower PDMS (polydimethylsiloxane) channels with a porous membrane in the middle resembling that of a sandwich. Polycarbonate membranes are the most commonly used membranes for this model, while polyethylene terephthalate and polytetrafluoroethylene are the alternatively used membranes [177]. Endothelial cells were seeded on the upper side of the membrane (i.e., the vascular compartment). In contrast, cells of the neurovascular unit, such as astrocytes, pericytes, or other brain cells, were cultured on the lower side of the membrane (i.e., the brain compartment). 

This design comes with some drawbacks as well. The first is inadequate cellular interactions between ECs and other cells seeded in the brain compartment due to the channel height. The other limiting factor of this design is the inability to perform real-time measurements of molecular transport, cellular growth, and high-resolution imaging due to the opaque nature of the membrane and cell seeding configuration. Substituting the membrane material with polyethylene terephthalate or polytetrafluoroethylene could help to resolve the issue of transparency [81]. 

A microfluidic assembly applying a sandwich design was constructed to study the transport mechanism of HDL-mimetic nanoparticles with apolipoprotein A1 (eHNP-A1) [11]. During fabrication, immortalized human microvascular endothelial cell (HBMEC), astrocytes and pericytes were used. They established that eHP-A1 nanoparticles employ scavenger receptor class B type 1 (SR-B1) for transport across the BBB through receptor-mediated endocytosis. With the use of block lipid transporter-1 (BLT-1), an inhibitor of SR-B1, the permeability of eHNP-A1 nanoparticles decreased drastically in perivascular space. The researchers established a significant claim of mechanistic transport of apolipoprotein A1-decorated nanoparticles through an in vitro model, which can further be extended to transport studies of other nanocarriers [11]. 

##### Parallel Design

The parallel design is another extensively used microfluidic assembly that features two parallel horizontal channels. The inner channel is plated with astrocytes, microglia, or neuronal cells, whereas the outer channel consists of ECs [178]. The distinguishing aspect of this design is the absence of the membrane found in sandwich designs and the presence of 3 µm perforations in the inner channel. These gaps in the inner channel serve as contact points between ECs and cells seeded within the inner channel to forge intercellular connections between them. The cells in the inner compartment can be plated as such or dispersed in hydrogels or collagen to closely mimic the basement membrane of the neurovascular unit [82]. 

Parallel designs are simple, avoiding the need for a separate membrane and preventing the sequential binding of individual layers during production. Compared to the sandwich design, this enables better cellular interactions [81]. This model is suited for acquiring high-resolution images or studying cellular behaviors. However, the thickness of the PDMS assembly, i.e., 50 µm, is much higher than the neurovascular unit’s basolateral membrane. 

Microfluidic chips were fabricated based on a parallel design [82]. It consisted of two concentric channels: vascular and tissue channels. The inner tissue channel was plated with primary rat astrocytes, whereas the vascular channel consisted of primary rat brain ECs. Zona occludin-1 signal, a marker of tight junction protein, was detected 5 days after the cell seeding. Permeability in this model was measured using an imaging technique. The fluorescent intensity of Texas Red 40 KDa Dextran from the vascular channel to the tissue channel was calculated at specific time intervals. The permeability was found to be 1.1 × 10^−6^ cm/s in this model, and this was closer to that found in neonatal rats of 0.4 × 10^−6^ cm/s but much lower than that observed in 2D transwell designed with similar cells (i.e., 7.5 × 10^−6^ cm/s) [82]. 

A microfluidic chip was designed based on a parallel model where two parallel microchannels coated with collagen IV–fibronectin were separated by a 50 µm thick, porous PDMS membrane [179]. The upper compartment was seeded with human iPSCs, and the lower compartment was seeded with primary human astrocytes and pericytes to build cellular connections. Immunocytochemistry performed on the barrier demonstrated the appearance of junction proteins such as occludin, whereas transcriptome analysis revealed the expression of endothelial transport proteins such as P-gp, BCRP, and several multidrug resistance-associated proteins. The TEER measurement reached as high as 1000 Ωcm^2^, validating the strong barrier formed. The permeability of fluorescein isothiocyanate tagged dextran (3 KDa) across the barrier was almost half compared to the barrier made using ECs alone [179]. 

##### 3D Tubular Structure Design

The chips fabricated using the photolithography process results in rectangular cross-sectioned channels using sandwich or parallel design. Due to this rectangular cross-section, erratic flow patterns and shear stress profiles occur, leading to changes in the behavior and morphology of ECs. Therefore, microfluidic channels with cylindrical geometry were designed, which ensures uniform shear stress distribution along the entire wall length. The tubular design is achieved either through hollow structures in a 3D hydrogel system or within hollow channels of microfluidic systems [177]. The ECs on the tubular architecture connect with astrocytes or pericytes plated on collagen. The extracellular matrix over the cylindrical tube aids in forming a natural basal membrane. Due to the extracellular gel matrix, the measurement of TEER is tough in this model, and also the collection of fluids for permeability analysis poses a challenge [81]. 

A microfluidic barrier model was constructed, incorporating a 3D tubular structure design [180]. A 120 µm channel was formed using a stainless steel needle in the collagen gel matrix interspersed with primary human pericytes. Human umbilical vein endothelial cells (HUVECs) were plated on the channel formed by the removal of needles. The permeability of this model was measured using Alexa-Fluor-488-labeled BSA after 6–7 days, and it was found to be 14.4 × 10^−6^ cm/s. The observation of relatively high permeability in this model compared to other 3D models discussed previously might be due to a lack of basal lamina in it.

##### Vasculogenesis Design

**Figure 6:**
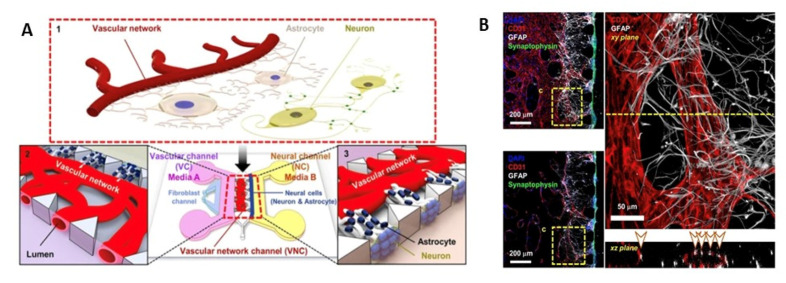
Self-assembled vasculatures of the BBB designed by the vasculogenesis approach. (**A**) The design depicts self-assembled endothelial cell vasculature in conjunction with astrocytes that harbors neuronal cells. The media for vascular channel and neural channel are supplied through side flanks. (**B**) Immunohistochemistry of vascular networks demonstrating astrocytes and vascular channel interface. Astrocytes end feet seen on the neuronal layer is spread at the surface of vascular channel which stops after the interface. (Source: Reprinted from Bang, S.; Lee, S.-R.; Ko, J.; Son, K.; Tahk, D.; Ahn, J.; Im, C.; Jeon, N.L. A low permeability microfluidic blood–brain barrier platform with direct contact between perfusable vascular network and astrocytes. *Sci. Rep.* **2017**, *7*, 1–10, [181], under an open access Creative Commons CC BY 4.0 license).

The previously described models consist of prefabricated channels through microneedles or microfluidic approaches to simulate endothelial lumen. Unlike these approaches, there are emerging devices that generate endothelial lumens within themselves de novo, termed vasculogenesis design (Figure 6). In this, the ECs are plated with fibrin hydrogels. Due to their inherent property to form lumen-like structures, the ECs outgrow and develop vascular structures. Astrocytes, pericytes, or neurons plated adjacent to the fibrin channels build connections with the endothelial layer to form a complete neurovascular unit. The cellular connections developed are superior to other models [177]. The blood vessel architecture generated by this design has ramified branching that prevents TEER measurement [81]. 

Hajal et al. [182] generated a BBB model using a vasculogenesis approach where two endothelial cell types (iPSC-derived endothelial cells (iPS-ECs) or primary human brain microvascular endothelial cells (HBMECs)), astrocytes, and pericytes in fibrin gel were used. The cells were plated in a central microfluidic gel channel flanked by two adjacent channels consisting of media. Due to their inherent ability to form lumen-like structures, the infused ECs in gel form tubular structures as they multiply and form connections with the existing astrocytes and pericytes around them, giving rise to a complete neurovascular unit. They either measured fluorescence signals or analyzed tracer molecules across the interstitial fluid for permeability determination. The relative permeability obtained with this model was close to that observed in animals. For 40 kDa, the permeability was 4.2 × 10^−8^ cm/s in this model compared to 1.4–1.9 × 10^−7^ cm/s in mice or rats. Similarly, the permeability of 10 kDa dextran was 3.1 × 10^−7^ cm/s compared to 1.7 × 10^−7^ cm/s observed in rats [182].

Researchers have developed barrier models for the penetration of fluorescently tagged polystyrene and polyurethane nanoparticles using a vasculogenesis technique [183]. Transferrin-conjugated nanoparticles demonstrated higher permeability across the BBB than plain nanoparticles for both types of nanoparticles. The PS-Tf nanoparticles had a permeability of 3.09 × 10 ^−7^ cm/s, almost double that observed in the case of plain nanoparticles, i.e., 1.64 × 10 ^−7^ cm/s. Similarly, the PU-Tf nanoparticles had significantly higher permeability of 3.70 × 10 ^−7^ cm/s compared to PU nanoparticles, i.e., 1.58 ×10^−7^ cm/s.

Straehla et al. [184] developed a barrier model using a vasculogenesis technique to analyze the penetration of liposomal nanoparticles. To generate a glioblastoma model in the microchip, the authors introduced spheroids that were separately formed on the central channel along with the typical cells of the neurovascular unit. The liposomal nanoparticles had a cationic core functionalized with the brain-specific ligand AP2 (Angiopep 2) for LRP1 receptor targeting, which is found to be overexpressed in glioblastoma. The change in permeability observed in AP2-decorated nanoparticles was significantly higher than in plain nanoparticles. Furthermore, research on nanoparticles using animal models confirmed the findings of in vitro barrier models. The researchers’ validation of in vitro experiments on animal models in this study indicates the closeness with which microfluidic models replicate the actual barrier. This gives us an important tool for screening many nanocarriers and drug molecules for their brain-targeting ability [184].

## 6. Conclusions and Future Perspectives

The role of in vitro BBB models is indispensable in expediting the CNS drug development process. Hence, the field has seen numerous approaches to design and study the barrier properties across the brain. The use of mouse, rat, bovine, or porcine cells is common in generating in vitro models. However, interspecies differences do not necessarily mimic the underlying human physiology, which might eventually affect the reproducibility and translational ability of these models. Incorporating human-derived cells is a better alternative. However, procuring human brain cells is always challenging, so hematopoietic stem cells derived from endothelial progenitor cells can be used as a model system in the future [185]. 

Both static and dynamic models generated using these cell types aid in the assessment of permeability across the brain or understanding barrier physiology, but we still are incapable of mimicking BBB properties in their entirety. Static models lack the element of shear stress in the models, while in dynamic models, the microvessel diameter is larger compared to cerebral vasculature, and it is also difficult to control the number of cells lining artificial microvessels. As a result, consistency and reproducibility of models remain challenges to overcome for high-throughput screening and effective clinical translation. Therefore, developing barrier models combining multiple approaches such as organoids on a microfluidic chip could be the future direction for better recapitulating the barrier in a reproducible manner [185]. Additionally, the barrier properties in healthy subjects are considerably different from those with CNS disorders as the neuroimmune crosstalk modulates the barrier properties in diseased states. Therefore, an unmet need exists to develop models simulating the barrier nature in the pathological state to increase the efficiency of in vitro models for disease-specific drug development. In silico models are increasingly being used to gauge the permeability of new therapeutics as these methods are economical, efficient, and can handle large samples; nevertheless, the validity of these results should always be confirmed with in vivo experiments [186]. 

## Figures and Tables

**Table 3:** Different markers used for the BBB permeability and their application with nanomedicine.

Marker	Merits	Demerits	References
Radiolabeled sucrose	Low molecular weight represents most of the small-molecule therapeutic drugs; offers quantitative permeability of the BBB	It can be metabolized	[149,150]
Sodium fluorescein	Low molecular weight, low limit of detection	Higher protein binding affinity than horseradish peroxidase	[144]
Horseradish peroxidase	Smaller protein, stable and relatively less expensive	Carcinogenic	[146]
Evan’s blue–albumin	Rapid, reliable, and highly sensitive assessment	It binds to albumin. Alteration of the structure in the presence of physiological buffers	[149,151]
Radiolabeled mannitol	Does not bind to plasma proteins, stable, uncharged. Does not interact with BBB transporters	It may contain lipophilic impurities	[15]
Trypan blue	Low toxicity	Binds to plasma proteins	[15]
Radiolabeled inulin	Does not bind to plasma proteins	It may contain lipophilic impurities	[15]
Dextran	It can be used for a wide range of molecular weight	Not suitable for low-molecular-weight permeability prediction	[15]
Albumin	It is used in the radiolabeled form, gives accurate quantification	Not suitable for low-molecular-weight permeability prediction	[15]

## Data Availability

Not applicable.
